# The History of Dentistry

**Published:** 1874-11

**Authors:** G. W. Field

**Affiliations:** Bd. Helvetique, Geneva, Switzerland


### THE HISTORY OF DENTISTRY.

This work, of which notice has been given heretofore, is in process of preparation for the press, as rapidly as completeness and precision will warrant. In the preparation of the work, a vast amount of material for consideration; and these-lection of the proper material and putting it in the proper form is a work of no small magnitude, far greater than we anticipated in the outstart. We have abundant evidence of the importance of such a work, and the interest the profession feel in it from the generous aid they are giving in the supply of material for its pages. But generous as this has been, there is doubtless a large amount of material, valuable and important for such a work, in possession of members of the profession. We trust that all such material will be sent to us at the earliest possible time, in order that all subjects may occupy their proper position in the work. Send to the editor of this journal.

Dr. George Field, of Switzerland, is preparing a list for publication of all the "Doctors of Dental Surgery" residing in Europe. He requests all dentists who have graduated in the States to send him, at their earliest convenience, their name and address and date of graduation, address

G. W. Field, D. D. S.,
Bd. Helvetique, Geneva, Switzerland.

